# Temperature and Agitation Are Highly Influential on Yield and Monodispersity of Self-Generated Carbon (SGC) Formed in Hydrothermal Carbonization Filtrate

**DOI:** 10.1021/acsenvironau.4c00150

**Published:** 2025-05-09

**Authors:** Alexandra Aveling, Kenneth G. Latham, Eva Weidemann, Stina Jansson

**Affiliations:** † Department of Chemistry, Umeå University, Umeå SE-90187, Sweden; ‡ Department of Chemical Engineering, Imperial College London, London, England SW72AZ, U.K.

**Keywords:** hydrothermal carbonization, carbon nano/microspheres, post-hydrothermal wastewater, aqueous co-product, carbon materials

## Abstract

Hydrothermal carbonization (HTC) offers significant potential for converting residual waste streams into advanced carbon materials with diverse applications. However, a key challenge in scaling up HTC is managing the large volumes of organic-rich filtrate produced during the process. Through a resting process, the filtrate can be repurposed to produce self-generated carbon (SGC). The spontaneously formed SGC exhibited a spherical morphology and low ash content, even when derived from complex, ash-rich precursors such as anaerobic digestate. SGC production from HTC filtrate may open up a new valorization route for industrial and municipal side-streams. In this study, we investigate how temperature, time, and agitation influence SGC yield, morphology, and particle size distribution. The cumulative yield was measured at intervals (days 2, 5, 7, 9, 26). The average cumulative yield after 26 days increased by 102 % at 50 °C compared to 20 °C, but decreased by 42 % at 4 °C. Agitated samples had the highest yield, increasing by over 260 % at 20 °C. The products showed variations in morphology and size distribution, with agitated samples producing more uniform and smaller particles. SEM imaging indicated a distinct product at 4 °C, with no visible spherical material being generated. Our results imply that changes in temperature and agitation are highly influential in the formation of SGC and may be used in optimizing product yield, sphere size and uniformity. The consistent formation rate over the 26-day period suggests that extending the experimental duration could further increase material yield. This is supported by mass balance calculations.

## Graphical abstract

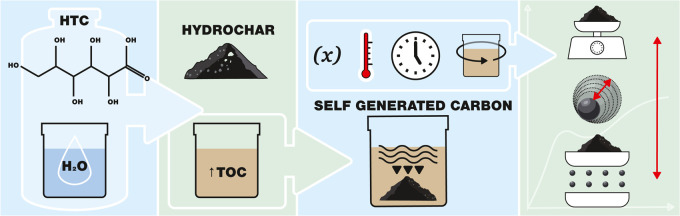

## 1 Introduction

After experiencing a resurgence in the late 2000s, hydrothermal carbonization (HTC) has emerged as a prominent technique for producing carbonaceous materials from wet biomass and waste streams.^1^ During HTC treatment, the feedstock is heated in a sealed vessel, with additional water provided if the inherent water content of the feedstock is insufficient. The organic material in the feedstock breaks down through a series of chemical reactions, resulting in the formation of a small quantity of gas, a carbonaceous solid (hydrochar), and a liquid fraction (sometimes referred to as HTC filtrate). The filtrate is rich in organic content and is commonly generated in large volumes.^2^ Using HTC, carbonaceous materials have been generated from a large variety of feedstocks, including crop and forestry residues,^3–7^ sewage sludge,^8–11^ industrial waste,^6,12,13^ and animal manure and food waste.^14–19^ These carbonaceous materials have been used as soil amendments, energy carriers^20^ and catalysts, as well as for carbon sequestration and remediation of pollutants in aqueous systems.^21,22^

The increased focus on HTC as a method of carbonization is predominantly driven by its requiring lower temperatures (i.e., 180–250 °C) than dry thermochemical conversion processes, and by the ability to directly carbonize and dope wet materials without pre-drying.^21^ However, the large volume of HTC filtrate generated in the process poses significant environmental and economic challenges, not only because efficient resource utilization assumes minimization of unused fractions but also because of its high content of dissolved organic carbon and chemical oxygen demand.

Studies indicate that HTC treatment of biomass residues yields net environmental benefits across most impact categories, but primarily for those unrelated to or only weakly impacted by the discharge of untreated filtrate.^3,16,23^ The production of filtrate is frequently cited as a significant bottleneck when it comes to the upscaling of HTC. One challenge is the insufficient knowledge about filtrate compositions due to current limitations in analytical techniques and inconclusive results.^24^ Attempts to use processes such as wet oxidation and nanofiltration to use or purify the filtrate have had varying outcomes. This is one of the factors preventing industrialization.^24^ The discrepancy between the state of knowledge of solid products and the state of knowledge of filtrate products underscores the need for further research to develop strategies to use the liquid by-product and so meet the demand for societal circularity. Such efforts will also support the sustainable production of the advanced carbon materials required for the next generation of clean catalysis, water treatment, structural reinforcement, and energy storage.^25,26^ Carbon microspheres, for example, are an ideal candidate for many advanced applications because of their unique properties such as uniformity, reduced packing density, and low surface free energy.^27^

The generation of high-purity carbon microspheres from HTC of sugar-based precursors was initially documented by Wang et al.^28^ However, obtaining high-purity carbohydrates to use as feedstock in HTC production of carbon spheres involves extraction from biomass, which is costly and resource-exhaustive.^29^ The increased focus on HTC processing of biobased waste streams may be a route to production of advanced carbon materials like carbon microspheres at a lower cost and with a smaller environmental footprint.

Over time, the method used to produce spherical carbon via HTC has been adapted to use different carbohydrate precursors.^4,29–32^ However, streamlined production is generally restricted by simultaneous solid–solid and liquid–solid conversion processes. This is particularly true for feedstocks containing both soluble and insoluble organic species, as seen in complex biomasses derived from residual waste streams. Solid–solid conversion typically produces a product that retains the macrostructure of the parent material.^5^ This poses a challenge, since producing spherical carbon materials depends primarily on liquid–solid conversion processes that allow morphological transformations.^32–34^ Consequently, complex feedstock mixtures containing both insoluble and soluble organic species result in hydrochars with a combination of carbon microspheres and macrostructures.^35,36^ The production of spherical carbon from such feedstock has so far required the use of additives or supplementary treatment.^4,15,20,32,33,37^

In 2021, we observed low-ash carbon microspheres, referred to as self-generated carbon (SGC), spontaneously forming from the hydrothermal supernatant from complex precursors.^35^ Unassisted generation of SGC would capitalize on the inherent separation between soluble and insoluble organic species, thus eliminating the need for prior extraction. It can be achieved by routine filtration of solid material (above a certain cut-off size) after HTC and retaining any remaining dissolved carbon in the filtrate for use in SGC generation. In our examination of SGC material, we generated microspheres from HTC filtrate originating from organic feedstocks such as anaerobic digestate, food waste, and horse manure.^35^ When allowed to rest, the SGC formed spontaneously from dissolved organic carbon in the HTC filtrate, without the use of additional chemicals or extraction steps.

Initial characterization of the SGC material indicated that it exhibits unique properties compared to hydrochar, for example, a notably low ash content and favorable properties for energy storage after activation.^35^ To the best of our knowledge, this is the first study to explore the influence of resting conditions on SGC formation by examining key factors such as temperature, time, and agitation. This investigation serves as a first step towards understanding the importance of these fundamental parameters. This will lay the foundation for efficient resource utilization of HTC feedstocks through production of a novel, waste-derived spherical carbon, with promising applications in advanced technologies. The aim of this study was to assess the selected conditions with regard to their influence on SGC yield, particle size distribution, and morphology. In this initial study, to limit the degree of chemical complexity in the HTC filtrate, we used glucose as the model precursor rather than more complex and inherently variable waste streams.^38^ Not only does the relatively uniform chemical structure of glucose facilitate well-controlled and reproducible experimental conditions, but its conversion under HTC conditions is well described in scientific literature. Therefore, using glucose as a model precursor, we here take the next step in exploring this novel material by examining the effects of temperature, time, and agitation on SGC yield, size distribution, and morphology.

## 2 Materials and Methods

### 2.1 Hydrothermal Carbonization of Glucose

Hydrothermal carbonization was conducted in a 20 L autoclave (Buchi AG) equipped with a programmable heating controller (Unistat 7305w HT) and water cooling. The autoclave was loaded with 1.8 kg glucose (Sigma-Aldrich, >99.5 GC) and 14 L tap water, and was heated to 250 °C for 3 h. This glucose-to-water ratio of approximately 1:7.8 falls within the range often reported in HTC research (1:1-1:20).^1–8,10–12,14,15,17–28^ The processing temperature was selected based on the maximum SGC yield observed at 260 °C by Latham et al.,^35^ slightly adjusted to 250 °C due to instrument limitations. After cooling overnight, the slurry was filtered in two steps: first through a coarse cotton cloth to remove most of the hydrochar, and then through a Whatman Grade 3 paper filter. The HTC heating profile is provided in the Supporting Information (Figure S1).

### 2.2 Self-Generation and Separation of Solid Carbon Material

The filtrate was split into multiple subsamples (6–8 aliquots per HTC run, 1.6–1.8 L each), weighed (at 20 °C), covered in aluminum foil to prevent exposure to light and limit evaporation, and left for up to 26 days at three different temperature conditions (4, 20, and 50 °C). All samples were filtered on day 2, 5, 7, 9, and 26 using a Whatman Grade 3 filter paper (Table 1), except for a separate set of samples that were left for 26 days before filtration (denoted “Single filt.” in Table 1). An additional set of samples was left on an orbital agitation table (IKA KS 260 Basic) at 30 rpm for continuous agitation before being filtered on day 26 (denoted “Agitated Single filt.” in Table 1). All samples were done in triplicate.

**Table 1:** Experimental Conditions of Self-Generated Carbon (SGC) Separation from Filtrate

	4 °C		20 °C		50 °C		agitated, 20 °C
days	multiple	single filt.	multiple	single filt.	multiple	single filt.	single filt.
2	*n* = 3		*n* = 3		*n* = 3		
5	*n* = 3		*n* = 3		*n* = 3		
7	*n* = 3		*n* = 3		*n* = 3		
9	*n* = 3		*n* = 3		*n* = 3		
26	*n* = 3	*n* = 3	*n* = 3	*n* = 3	*n* = 3	*n* = 3	*n* = 3

The collected SGCs were freeze dried on the filter paper for at least 3 days before weighing to determine the mass of self-generated carbon in relation to time and initial weight of filtrate. The SGC yield was defined as the ratio of the total weight of SGC after all filtrations to that of the initial weight of the filtrate (eq 1). It was expressed as a cumulative yield.

1 $$\mathrm{SGC}\;\mathrm{yield}=\frac{m_{\mathrm{SGCday}2}+m_{\mathrm{SGCday}5}+m_{\mathrm{SGCday}7}+m_{\mathrm{SGCday}9}+m_{\mathrm{SGCday}26}}{m_{\mathrm{filtrate},\mathrm{initial}}}$$

### 2.3 Photographic Documentation

The samples were photographed using a Nikon D5000 camera with an AF-S NIKKOR 18–55 mm 1:3.5–5.6G lens. To ensure consistent light conditions, the samples were placed in a light box with the camera at a fixed distance. Image settings were manually set (1/30, f 5.0, ISO 200, 36 mm).

### 2.4 Scanning Electron Microscopy (SEM)

The dried SGC material was attached to sample holders using carbon tape and blown with high-pressure N_2_ gas to remove any loose particles. Subsequently, samples were coated with a 10 nm Pt layer to prevent electric charge build-up. To determine morphology, SEM analysis was performed using a ZEISS EVO SEM operated in low vacuum mode.

SEM imaging was used to determine particle size distribution. Between 200 and 500 sphere diameters were assessed for each condition, depending on the number of particles visible in the images. For this, the software ImageJ 1.54k was used.

### 2.5 Ash Content

Ash content was assessed by placing a previously weighed amount of material on a Petri dish in a furnace (Nabertherm, Controller B410). All samples were done in triplicate. The furnace was heated to 550°C overnight, after which the remaining material was weighed again.

## 3 Results and Discussion

### 3.1 Influence of Temperature on Formation and Morphology

Three different temperature conditions (4, 20, 50 °C) were selected to assess the impact of temperature on the generation of SGC. In each HTC run, around 750–800 g of hydrochar was generated. According to data by Ischia et al.,^39^ under our chosen operational conditions and for glucose feedstock, the hydrochar is estimated to have a 70 % carbon content. Starting with an initial C content of 720 g in the glucose precursor, approximately 300 g of C (calculated based on data by Ischia et al.,^39^ see Figure S2) was not recovered in the hydrochar. It is estimated that 1–3 % of this carbon was present in the gas fraction.^39^ The remaining carbon likely remained as dissolved species in the filtrate.

The average cumulative yield of SGC was initially within the same range for the 4 °C samples and the 20 °C samples (Figure 1). However, at and after an extended resting time of 26 days, the cumulative SGC yield at 20 °C reached levels almost 70 % higher than those observed at 4 °C (358 versus 208 mg/L). To assess the influence of increased temperature, a set of samples was placed in a heating cabinet at 50 °C for 26 days, with filtrations after 2, 5, 7, 9, and 26 days. The high-temperature conditions doubled the average cumulative SGC yield in the samples, with 724 mg formed after 26 days at 50 °C compared to 358 mg at 20 °C. The observed differences in cumulative yield support our hypothesis that the formation of SGC is temperature-dependent.

**Figure 1:**
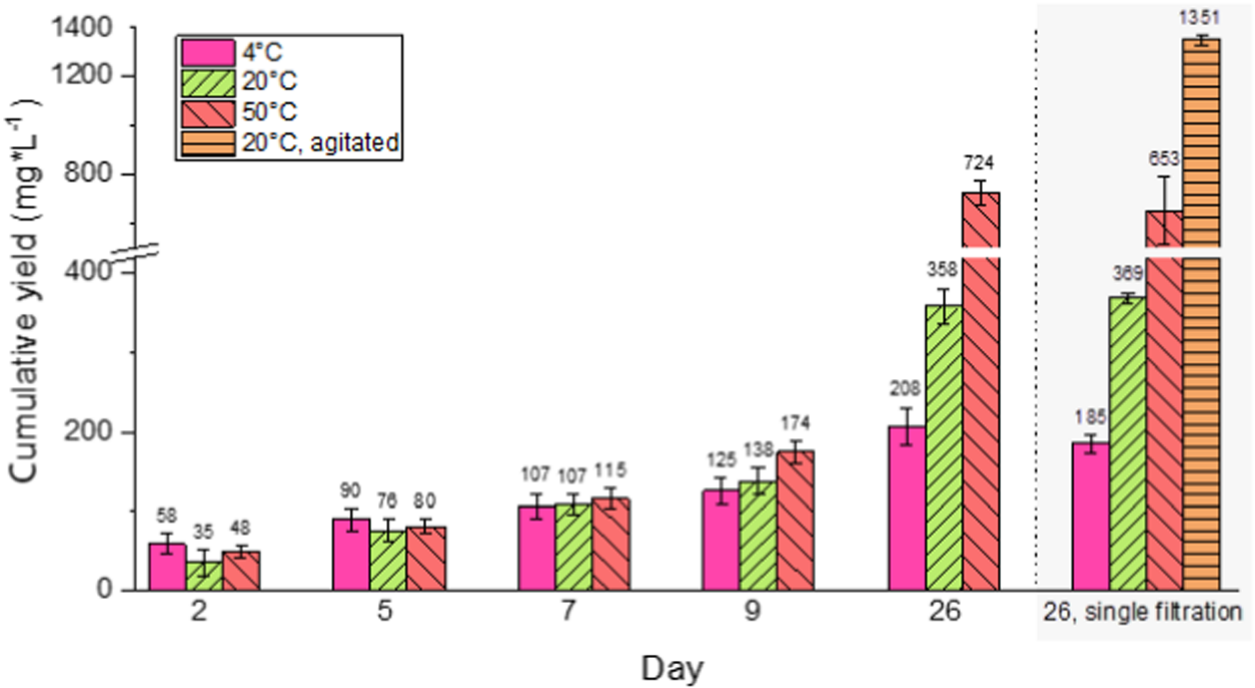
Cumulative SGC yield (average of three samples) with error bars showing ± one standard deviation.

A yield of up to 724 mg of SGC per L of filtrate makes up a small fraction of the approximately 300 g of C estimated to be dissolved in the total volume of the filtrate (see Figure S2), indicating that there are still substantial concentrations of dissolved carbon remaining even after 26 days. The residual carbon content is likely influenced by the presence of carbonaceous colloidal nanoparticles in the solution, first identified by Xu et al.^40^ In addition, dissolved volatile fatty acids, sugars, and aromatic compounds may also contribute to the remaining carbon content.^41,42^ Additionally, the relatively constant and linear SGC formation rate (*R* ^2^ = 0.968 at 4 °C; *R* ^2^ = 0.999 at 20 °C; *R* ^2^ = 0.985 at 50 °C, see Figure S3) until day 26 indicates that extending the resting time of the samples could add to the total yield.

The cumulative yields of the multiple and single filtration samples were compared to examine the importance of the frequency of filtration in the yield. The repeated removal of the material (via multiple filtrations) did not lead to statistically significant differences in yield for the three temperature conditions (Figure 1). This indicates that SGC solubility has a limited impact on formation, that is, its formation is not likely to depend solely on a solubility equilibrium.

The visual appearance of the SGC was also impacted by the formation temperatures. For example, at 20 °C, the SGC fell out in the form of a porous, non-reflective powder (Figure 2D), while the SGC formed at 4 °C appeared as droplets of a dense, shiny, tar-like substance that was sticky and highly viscous, with a pungent, sweet, burnt smell. SEM images (Figure 2E,2F) reveal that at 20 °C the HTC filtrate formed SGC with smooth, nearly discrete, spheres on the micron scale (up to 9 μm). The formation and spheroidization of the material are likely driven by an emulsification process involving colloidal nanoparticles. During this process, the hydrophobic nature of the carbon components reduces surface tension, facilitating the self-assembly of carbon into spherical structures.^39,43^ Spherical micro carbons are highly valued in energy-related applications due to their ability to facilitate uniform ion flux and efficient charge transfer in sodium-ion batteries, as highlighted by Ischia et al.^39^ Furthermore, their effectiveness in water remediation is significantly higher, with adsorption capacities nearly double those of non-spherical biochar, as reported by Tran et al.^44^ Some of these spheres had fragments attached to their surfaces, possibly remnants from the physical removal of interconnected spheres during filtration or when N_2_ gas was used in preparing the SEM sample. The spherical morphology of the SGC did not resemble the glucose precursor (Figure S4), and is therefore likely to have been formed in a liquid–solid conversion process occurring after the initial hydrochar filtration. Similarly, all hydrochar generated from HTC of soluble carbohydrate feedstocks originates from liquid–solid conversion processes.^5,17,39^

**Figure 2:**
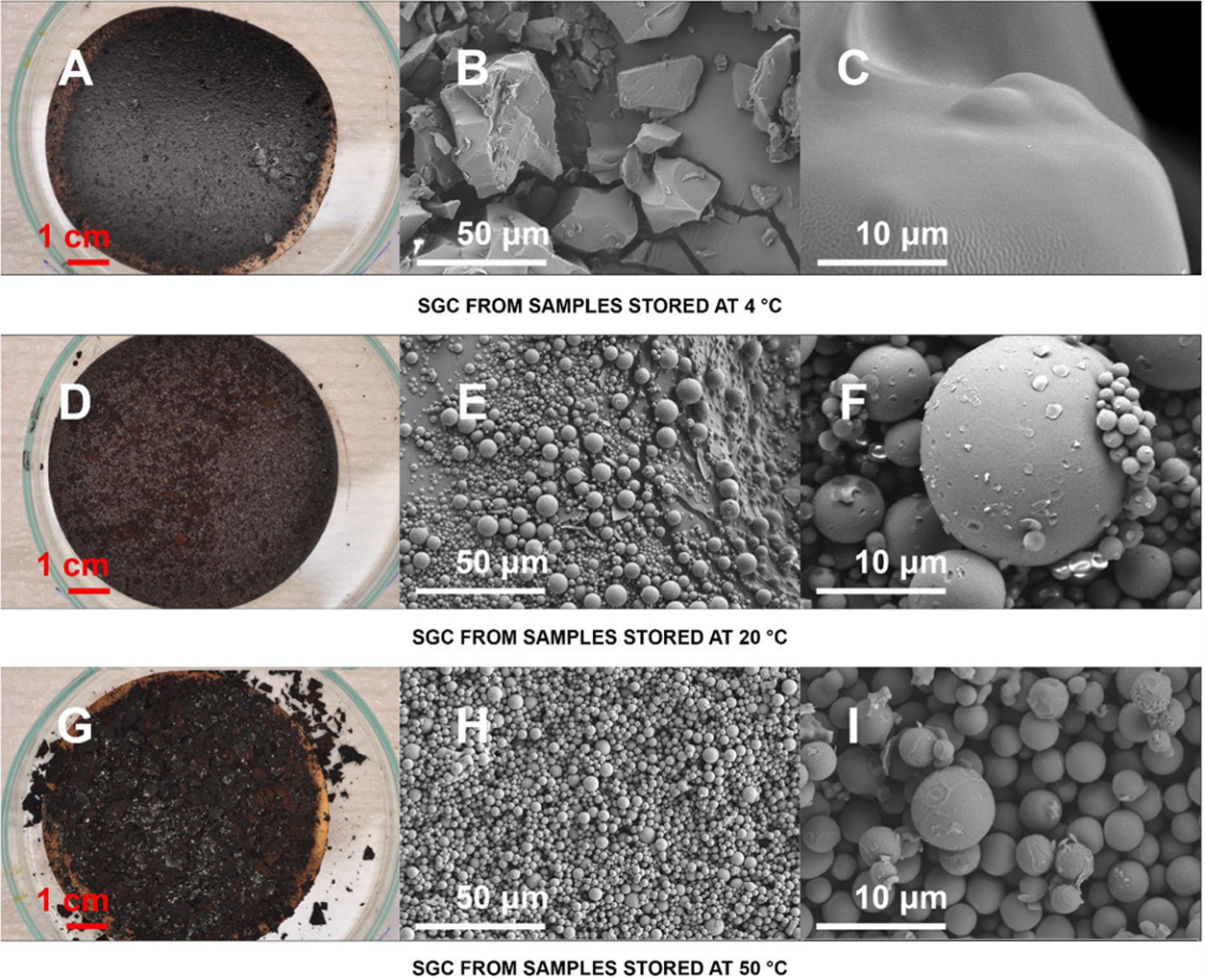
Photos and SEM images of SGC formed from filtrate maintained at different conditions (4, 20, and 50 °C).

The SGC formed at 50 °C presented a different morphology from that of SGCs generated at 20 °C, as observed in Figure 2H,2I. In the 50 °C samples, large aggregates of spheres were visible in SEM as well as in the photographic documentation (Figure 2G). These aggregates were composed of individual SGC spheres. The particle size distribution exhibited a distinct right-skewed distribution, with a prominent peak at 0.7 μm, suggesting a preference for smaller particles (Figure 3). The particles formed at 20 °C aggregated in smaller macrostructures, with the individual sphere size distribution peaking at 1.7 μm, with an additional peak at 2.1 μm, and some spheres reaching sizes up to 8–9 μm. We hypothesize that the narrower particle distribution of the SGC spheres formed at 50 °C may be attributed to rapid stabilization of colloidal spheres, which prevents coalescence and promotes monodispersity by stabilizing particles early. Higher temperatures accelerate reaction rates (e.g., oxygen reactions and dehydration) which can lead to rearrangement or regeneration of functional groups that aid in cross-linking. Dehydration of glucose produces hydroxyl, carbonyl and carboxyl groups, which play a key role in facilitating hydrogen bonding and enhancing intermolecular interactions.^45^ These functional groups can also contribute to cross-linking through mechanisms such as condensation reactions or nucleophilic addition, resulting in the formation of new covalent bonds.

**Figure 3:**
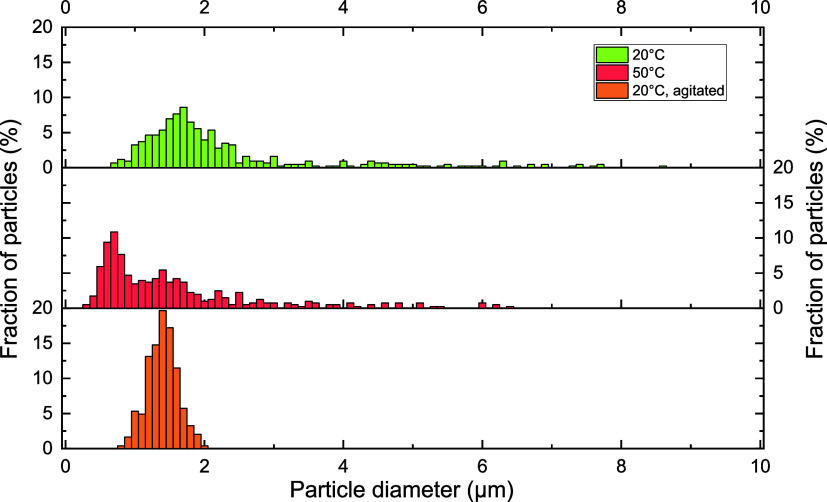
Relative size distribution of samples stored at 20 and 50 °C, and agitated at 20 °C, as estimated by analyzing the SEM images.

Monodispersity may also arise from simultaneous nucleation and uniform growth due to high energy or temperature-driven processes. Ostwald ripening, where smaller droplets dissolve and contribute to larger ones, is mitigated by rapid stabilization.

Parts of the aggregates formed at 50 °C are covered by an additional, distinct layer of material (Figure S5); seemingly, the same material is visible in much smaller masses between individual spheres.

By contrast, the material formed at 4 °C consists of amorphous particles with smooth surfaces and a wide range of sizes. On the basis of its smell and viscosity, we hypothesize that the amorphous material formed over time at 4 °C is a tar-like substance. This phenomenon might resemble the precipitation of dissolved tar when the temperature drops below its softening point.^42,43^ In a study by Yuan et al.,^43^ a method for producing carbon spheres from tar was outlined, utilizing spheroidization and stabilization pathways. This approach, which incorporated emulsion polymerization alongside simultaneous gas bubbling, linked the spheroidization to specific emulsion conditions.

In an industrial context, tar formation could be problematic as it could cause congestion, corrosion, and operational issues in downstream processes.^8,46,47^ There is a clear need for more investigation to determine the optimum temperature for SGC formation while minimizing tar formation.

### 3.2 Agitation

A crucial difference between the formation of hydrochar and SGC is the continuous mixing during HTC processing, facilitated by the integrated stirring in the reactor. To date, SGC formation has occurred without continuous agitation. In this paper, the effect of agitation on SGC generation was investigated by subjecting one set of the 20 °C samples to continuous movement by placing them on an orbital agitation table. The yield of the agitated samples was 1351 mg/L after a single filtration at 26 days, compared to 369 mg/L for the corresponding 20° C samples (i.e., those kept without agitation), representing a more than 260 % increase in yield (Figure 1). We hypothesize that this increase in yield may be attributed to a combination of an increase in the collision frequency, the kinetic energy of the reactants, and oxygen availability.

Compared to SGC formed under undisturbed conditions, the SGC spheres that formed during agitation exhibited greater uniformity, as evident from the size distribution analysis (Figure 3). Specifically, SGC formed without agitation exhibited a size distribution extending up to 9 μm, whereas when agitation was employed, the size distribution narrowed significantly, with a maximum observed diameter of 2 μm. In the case of the agitated sample, the size distribution followed a normal distribution with the peak at 1.4 μm, in comparison to the non-agitated sample peak at 1.7 μm. This is a critical finding, for it suggests that the spherical size could be manipulated and potentially tailored using agitation.

Hydrochar is produced during agitation, although it is induced by stirring rather than orbital movement, which is likely to lead to different levels of mechanical stress being applied. In addition, HTC and SGC formation takes place at substantially different concentrations and possibly different compositions of organic species within the filtrate. Stirring the reactor during HTC has been shown to induce notable changes in the morphology of the resulting hydrochar.^32^ According to their study, when formed during stirring, hydrochar produced from fructose consisted of agglomerations of carbon spheres of a strongly disrupted morphology, along with small carbon spheres in the bulk solution. Our SEM imaging of the hydrochar (Figure 4) shows a very similar morphology to that described by Jung et al.^32^ in terms of being agglomerated and disrupted. On the other hand, hydrochar formed in non-stirred HTC processes was described by Jung et al.^32^ as having an exhaustive spherical morphology. They attributed the deformation of stirred spheres to mechanical pressure prior to solidification. The SGC formed during agitation did not show this notable agglomeration (Figure 4). This observation may suggest that SGC formation follows a different path from hydrochar, originating in a solid state from the onset of growth and thus retaining its spherical shape. Importantly, both of the agitated materials (i.e., both hydrochar and agitated SGC) exhibited a remarkable degree of monodispersity (Figure 3) compared to non-agitated SGC samples.

**Figure 4:**
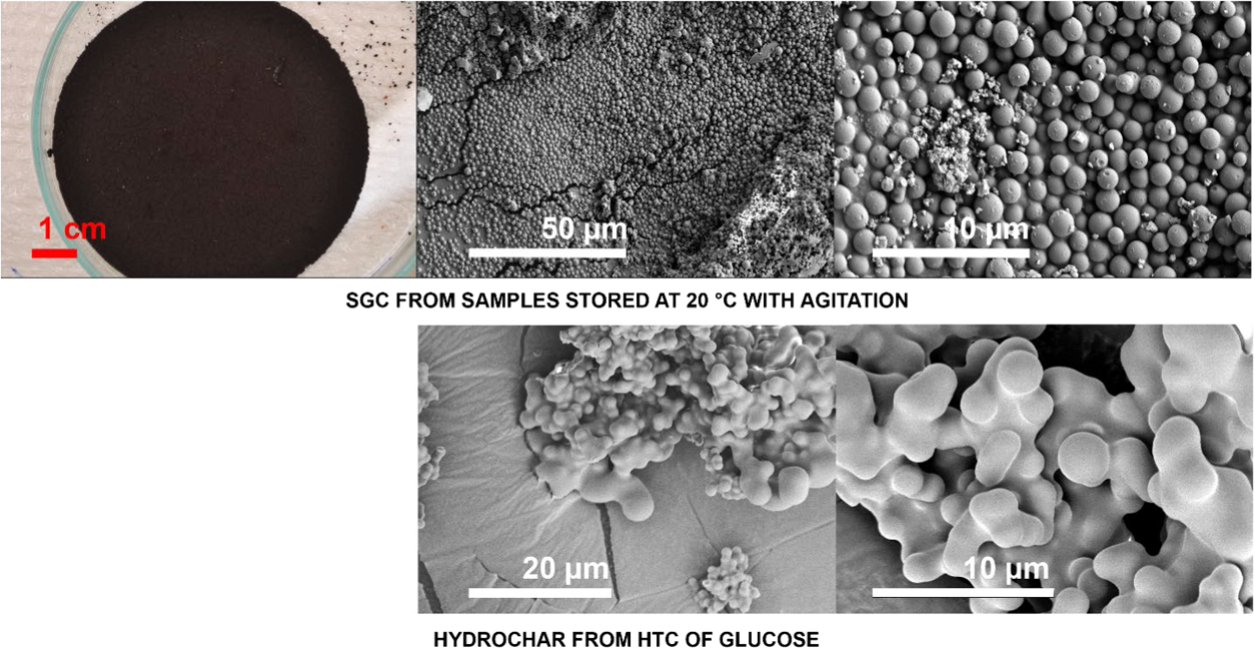
Photos and SEM images of SGC formed from filtrate maintained at 20 °C with agitation and from hydrochar from HTC of glucose.

### 3.3 Ash Content

The ash content of all materials, that is, the glucose precursor, the hydrochars, and the SGCs, was determined using LOI550 and compared to the data reported by Latham et al.^35^ All SGCs in this study showed a low ash content (<0.3 %), consistent with the minimal ash content of the initial glucose feedstock (0.01%). Interestingly, hydrochar from glucose showed slightly higher ash content than the untreated glucose feedstock. This is attributed to insoluble impurities in local tap water that were concentrated during the solid–solid conversion process. The tap water in this municipality has been found to contain metal ions at concentrations up to 20 mg/L, sometimes exceeding 50 mg/L cumulatively (Vakin, 2023). The ash content of the SGC represents a small fraction (1–4 %) of the total metal content in the corresponding volume of tap water used during the HTC runs, ranging from 0.64 mg/L (SGC formed at 20 °C over 26 days) to 1.67 mg/L (SGC formed at 50 °C over 26 days). Hence, it is likely that the ash content was notably influenced by the incorporation of metals into the SGC. Naturally, with increasing complexity of feedstocks, higher concentrations of dissolved metals may be introduced. However, in the study by Latham et al.,^35^ SGC produced from anaerobic digestate demonstrated a much lower ash-content (3.1%) than both the feedstock (25.2%) and the hydrochar (41.2%).

## 4 Conclusions

The average cumulative yield of SGC after 26 days was 42 % less when stored at 4 °C than when stored at 20°C. However, the cumulative yield increased by 102 % when the sample was kept at 50 °C. An even higher yield was found for the agitated samples, with an average increase of over 260%. The SGCs formed under different conditions also displayed varied morphology and size distributions, with the most distinct difference being the monodispersity and reduced size of the SGCs formed during agitation. SEM imaging also revealed a seemingly distinct product formed at 4 °C, with no visible spherical material being generated.

The calculated mass of carbon species not accounted for by the hydrochar suggests that a substantial amount of organic content remains in the filtrate, and that the SGC formed represents only a small fraction of the original content. Future research should expand the investigated time frame to examine SGC formation beyond the 26-day time frame, and should also embark on detailed characterization of specific carbon species before and after SGC generation to identify those that contribute to SGC formation.

Furthermore, to better understand the composition and formation of SGC, extensive chemical and physical characterization of its bulk and surface composition is required. Incorporating more complex HTC precursors such as forestry and agricultural residues will be crucial for evaluating the effects of different feedstock on SGC properties. The effect of agitation needs to be further explored to determine which factors affect yield and size distribution, for agitation induces several changes. These factors include the air/oxygen supply, oxidizing agents, and the effect of mechanical movements/collision frequency.

## Supplementary Material



## Notes

Average heating profile of HTC runs (Figure S1); calculation of estimated carbon content in liquid fraction (Figure S2); formation rate of SGC from filtrate at different temperatures (Figure S3); and SEM of untreated glucose precursor (Figure S4) (PDF)
