# Supplementary material for: Temperature and Agitation Are Highly Influential on Yield and Monodispersity of Self-Generated Carbon (SGC) Formed in Hydrothermal Carbonization Filtrate
Source: ACS Environ Au. 2025 May 9;5(4):387–94. doi: 10.1021/acsenvironau.4c00150 (PMC12272275; doi:10.1021/acsenvironau.4c00150)
Supplement: Supplementary file 1 [file vg4c00150_si_001.pdf]

Temperature and agitation are highly influential on yield and monodispersity of self-generated carbon (SGC) formed in hydrothermal carbonization filtrate

Alexandra Aveling[a], Kenneth G. Latham[a,b], Eva Weidemann[a] and Stina Jansson[a]*

[a]*Department of Chemistry, Umeå University, SE-901 87 Umeå, Sweden;* [b]*Department of Chemical Engineering, Imperial College London, London, England, UK SW7 2AZ*

* Department of Chemistry, Umeå University, SE-901 87 Umeå, Sweden, Stina.Jansson@umu.se

Supplemental Information

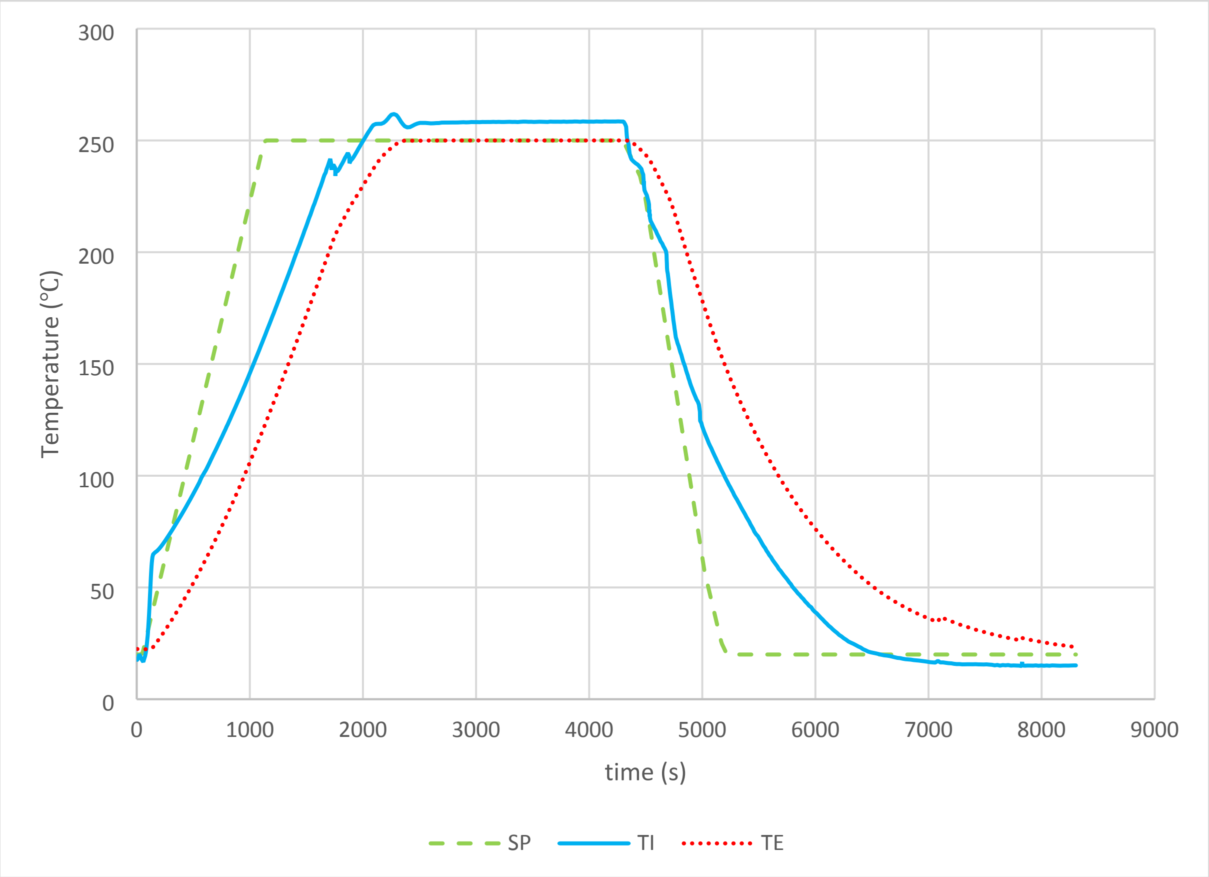

Fig. S1. Average heating profile of HTC runs. SP: set point; TI: internal temperature; TE: external temperature.

Temp (℃)	Time (h)	Mass Yields (%)		
		Solid	Gas	Liquid*
**250**	3	48.0	8.2	43.7

Mass Yields according to Ischia et al.

1800 g glucose corresponds to 720 g of C

720 g * 0.437 = 315 g

Fig. S2. Calculation of estimated carbon content in liquid fraction.

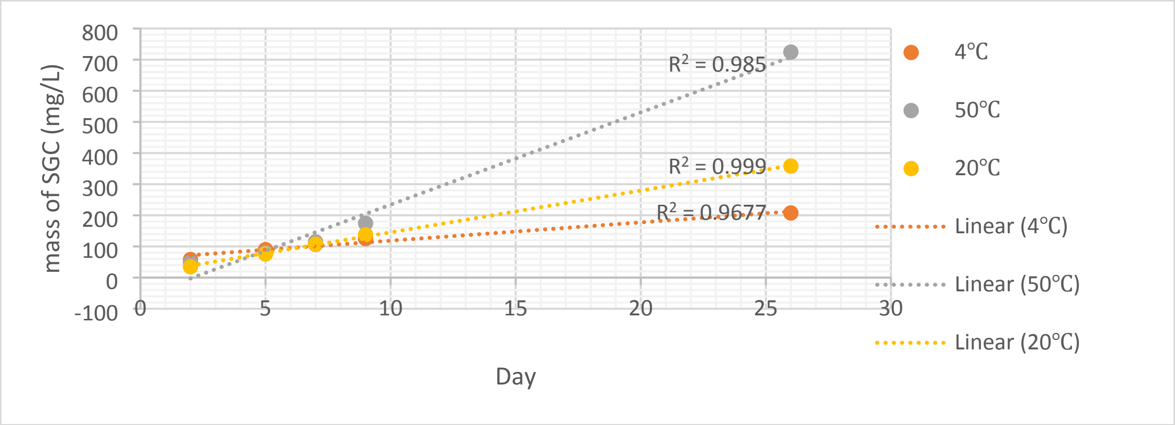

Fig. S3. Formation rate of SGC from filtrate at different temperatures (4 °C, 20 °C, and 50 °C).

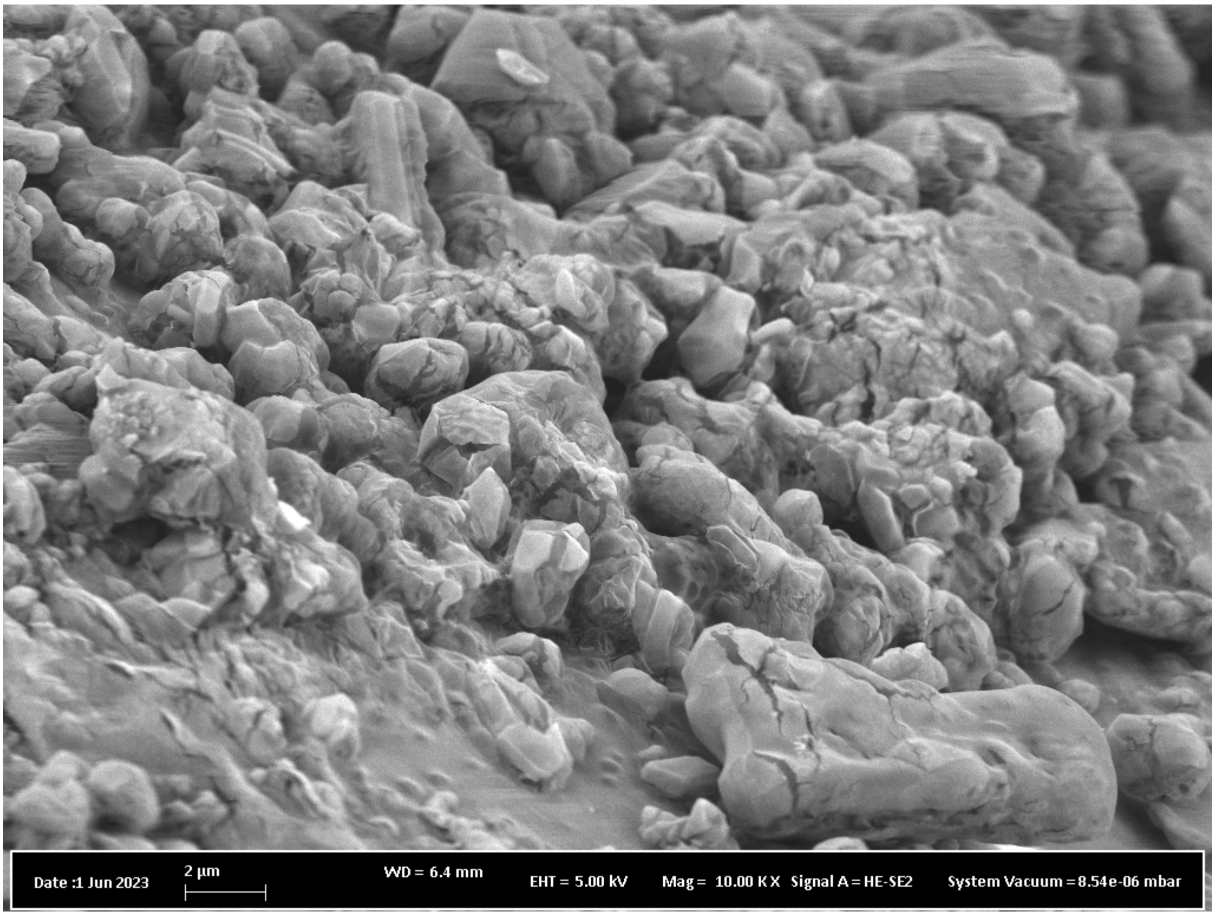

Fig. S4. SEM of untreated glucose precursor.

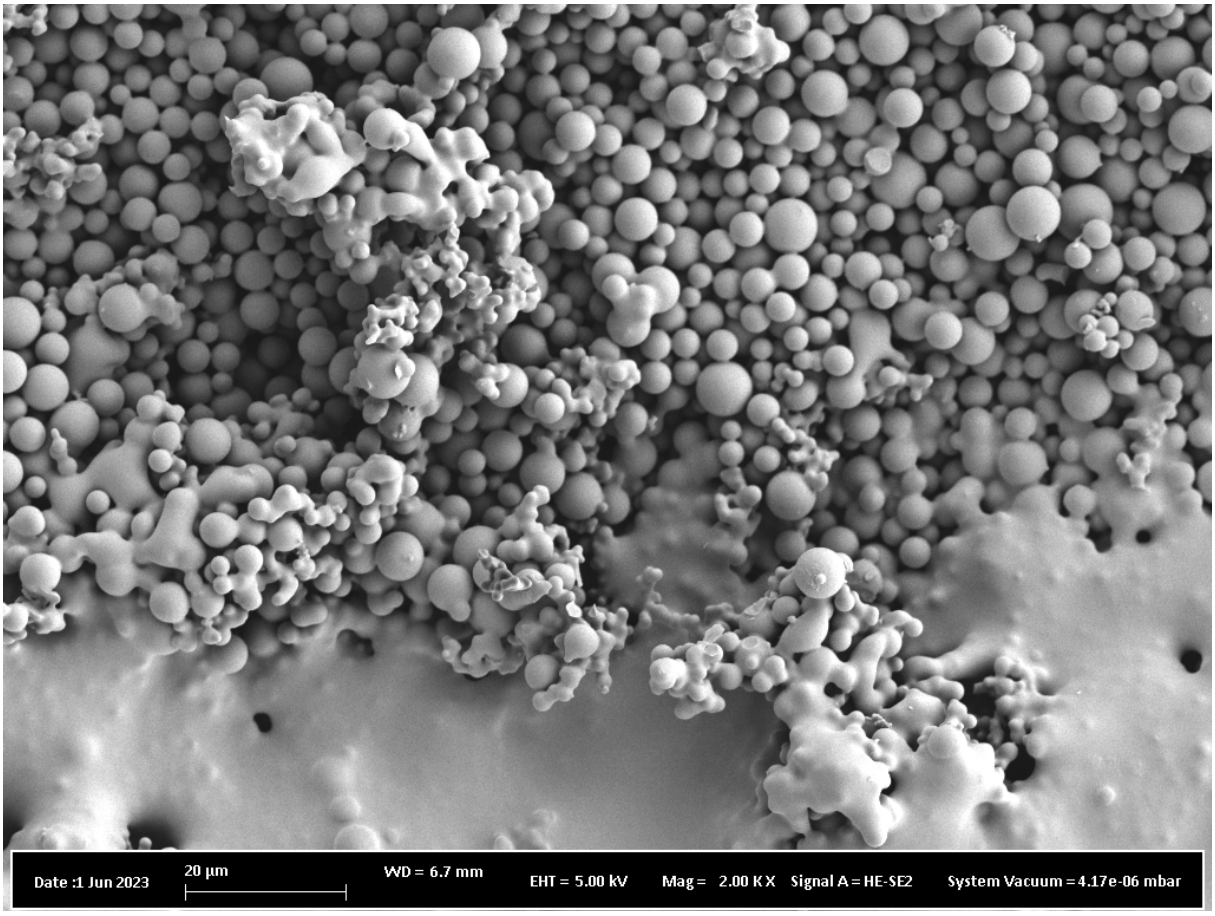

Fig. S5. SEM of SGC sample produced from filtrate kept at 50℃, with visible coating.
